# Antimicrobial Mechanisms of Macrophages and the Immune Evasion Strategies of *Staphylococcus aureus*

**DOI:** 10.3390/pathogens4040826

**Published:** 2015-11-27

**Authors:** Ronald S. Flannagan, Bryan Heit, David E. Heinrichs

**Affiliations:** 1 Department of Microbiology and Immunology, the University of Western Ontario, London, ON N6A 5C1, Canada; E-Mails: rflanna2@uwo.ca (R.S.F.); bheit@uwo.ca (B.H.); 2 Centre for Human Immunology, the University of Western Ontario, London, ON N6A 5C1, Canada

**Keywords:** macrophage, phagocytosis, immunity, *Staphylococcus*, anti-phagocytic, immune evasion, nutritional immunity

## Abstract

Habitually professional phagocytes, including macrophages, eradicate microbial invaders from the human body without overt signs of infection. Despite this, there exist select bacteria that are professional pathogens, causing significant morbidity and mortality across the globe and *Staphylococcus aureus* is no exception. *S. aureus* is a highly successful pathogen that can infect virtually every tissue that comprises the human body causing a broad spectrum of diseases. The profound pathogenic capacity of *S. aureus* can be attributed, in part, to its ability to elaborate a profusion of bacterial effectors that circumvent host immunity. Macrophages are important professional phagocytes that contribute to both the innate and adaptive immune response, however from *in vitro* and *in vivo* studies, it is evident that they fail to eradicate *S. aureus*. This review provides an overview of the antimicrobial mechanisms employed by macrophages to combat bacteria and describes the immune evasion strategies and some representative effectors that enable *S. aureus* to evade macrophage-mediated killing.

## 1. Introduction

*Staphylococcus aureus* is a prolific human and animal pathogen that is a global cause of morbidity and mortality. Indeed, deaths attributed to *S. aureus* infection in the United States alone now approach mortality rates associated with HIV/AIDS and tuberculosis, emphasizing the severity of *S. aureus* infection as a health care threat [1,2,3]. While *S. aureus* was previously recognized as a common cause of nosocomial infection, some strains have a propensity to disseminate between otherwise healthy individuals giving rise to community-acquired infections [4]. Further amplifying the gravity of *S. aureus* infections is the emergence of multi-drug resistant strains such as methicillin-resistant *S. aureus* (MRSA) that can demonstrate enhanced infectivity and virulence [5,6]. Incredibly, these bacteria can colonize virtually every tissue in the body causing pathologies varying from minor to severe skin and soft tissue infections to fatal invasive diseases such as necrotizing pneumonia, osteomyelitis and sepsis [7,8,9]. The success of *S. aureus* as a pathogen can, in part, be attributed to its vast repertoire of virulence determinants that enable the bacteria to efficiently extract nutrients from its host and to thwart both innate and adaptive immune attack by the host.

**Figure 1:**
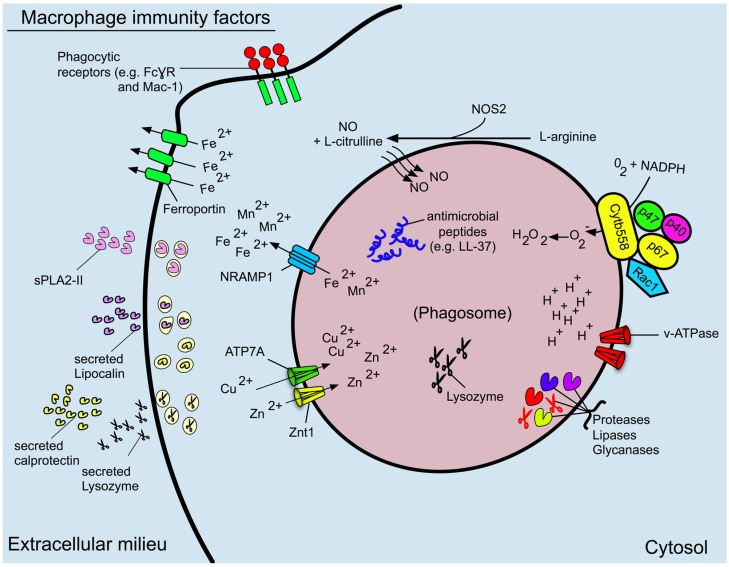
An overview of the antimicrobial mechanisms of macrophages and *S. aureus* immune evasion effectors. Shown is a summary of the anti-microbial functions of a macrophage. Several factors (e.g., NADPH oxidase, NRAMP-1, and cathepsin proteases) operate in the phagosome lumen while others, such as lipocalin, secreted lysozyme and sPLA2-II, operate in the extracellular milieu. Not represented here is the process of phagocytosis that, although is essential to macrophage-mediated killing of bacteria, is not in of itself microbicidal. Moreover, the formation of mETs has been omitted but may prove to be an under-appreciated antimicrobial mechanism of the macrophage. Abbreviations: Fe^2+^, ferrous iron; NRAMP-1; natural resistance-associated membrane protein 1; NOS2, nitric oxide synthase 2; NO; nitric oxide, O_2_^−^; superoxide; H^+^, protons; v-ATPase, vacuolar ATPase; H_2_O_2_, hydrogen peroxide; Zn^2+^, zinc; Cu^2+^, copper. Znt1, zinc transporter; ATP7A, copper transporter.

Professional phagocytes such as macrophages and neutrophils comprise an integral facet of the host immune response and the interaction of neutrophils with *S. aureus* has been intensely characterized (for a recent review see [10]). Remarkably *S. aureus* can withstand neutrophil mediated killing, an impressive feat considering the potent microbicidal capacity of the neutrophil. In contrast, the interaction of *S. aureus* with macrophages has been less scrutinized, however investigation of this interaction has garnered recent attention. Like neutrophils, macrophages are professional phagocytes that are equipped with an impressive armamentarium of antimicrobial effectors and thus represent an important component of the innate immune response. Furthermore, macrophages can shape adaptive immunity through presentation of antigens that are derived from fluid phase uptake and phagocytosis of microbial prey [11,12]. Given the immune functions of the macrophage it stands to reason that evasion of macrophage-dependent killing is required to successfully establish and maintain infection. In this review we present an overview of the antimicrobial mechanisms of macrophages and describe some salient examples of bacterial evasion strategies and effectors that are employed by *S. aureus* to counteract host macrophages (for an overview of macrophage defenses, see Figure 1).

## 2. The Macrophage: A Sentinel of Immunity

Macrophages comprise complex populations of cells that can either be self-renewing tissue resident cells or can be derived from circulating monocytes in response to physiologic stimuli [13,14,15]. Remarkably macrophages can display a spectrum of functional states that are determined by the presence of signals (e.g., cytokines) in the local microenvironment of the cell. This functional plasticity has given rise to the notion of macrophage polarization and is readily demonstrated *in vitro* where monocytes, in the presence of GM-CSF and inflammatory stimuli (e.g., IFNγ and LPS), give rise to so-called microbicidal M1 macrophages whereas culture of monocytes with M-CSF and IL-4 give rise to M2 macrophages that demonstrate functions associated with tissue remodeling and repair [16,17,18]. While these polarized states are readily obtained *in vitro* detection of their existence *in vivo* is less obvious and, presumably, this is explained by that fact that in an *in vivo* setting, macrophages exist on an ever-evolving continuum of these extremely polarized states because they continuously integrate gradients of environmental cues. Regardless of the existence of heterogeneous macrophage populations these cells are all unified through their capacity to combat infection, albeit with variable efficiency, because they are endowed with the innate ability to ingest particulates, such as bacteria, through phagocytosis.

## 3. Phagosome Formation and Maturation

**Figure 2:**
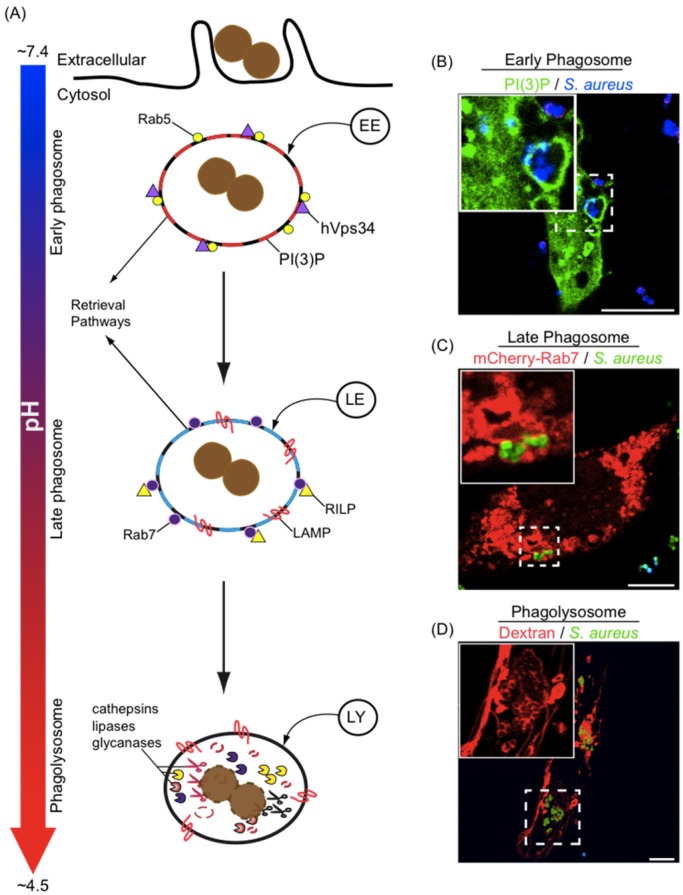
Maturation of phagosomes in macrophages. In (**A**) the maturation pathway that a newly formed phagosome typically follows is depicted. Nascent phagosomes interact sequentially with compartments of the endo/lysosomal network, giving rise to phagosomes that, at each maturation stage, possess biochemically distinct properties. Phagosomes can be classified as early phagosomes, late phagosomes, or phagolysosomes and the presence of specific markers aid in defining the state of phagosome maturation. Early phagosomes are marked with the small GTPase Rab5, the phosphatidylinositol 3 kinase Vps34, and the phosphoinositide PI(3)P. Late phagosomes are decorated with LAMP-1/2, Rab7, RILP and are devoid of PI(3)P and Rab5. Finally, phagolysosomes that are formed by fusion between late phagosomes and lysosomes, are enriched with hydrolytic enzymes (e.g., proteases, glycanases, and lipases) and are markedly acidic; In (**B**–**D**) representative laser scanning confocal micrographs depicting RAW macrophages containing *S. aureus* USA300 that reside in early phagosomes, late phagosomes, or in phagolysosomes, respectively, are shown. *S. aureus* in green are expressing GFP and *S. aureus* in blue were stained with eFluor670; In (**B**) accumulation of PI(3)P is detected by expression of the lipid biosensor 2xFYVE-GFP; In (**C**) the distribution of Rab7 expressed as a mCherry-Rab7 fusion protein is shown; In (**D**) dextran pulse chase experiments were performed to label lysosomes with TMR-dextran (10 kDa) prior to phagocytosis of *S. aureus*. For each micrograph the hashed box demarcates the region of the micrograph presented in the inset; Note in (**D**) the GFP channel was omitted from the inset to clearly show the accumulation of dextran around each coccus that appears as a void in the fluorescence. Bars equal ~10 μm. Abbreviations: PI(3)P, phosphatidylinositol-3-phosphate; LAMP, lysosome-associated membrane proteins; RILP, Rab7 lysosomal interacting protein; GFP, green fluorescent protein; TMR, tetramethylrhodamine.

Phagocytosis is a receptor-mediated process whereby macrophages ingest large particulate antigens (≥0.5 μm) into a membrane bound vacuole termed the phagosome [19,20,21]. Phagosome formation in and of itself is not microbicidal because the lumen of the nascent vacuole is a reflection of the fluid phase outside the macrophage and the limiting phagosomal membrane is derived directly from the cell membrane, however, with rapid succession the nascent phagosome undergoes significant biochemical remodeling revealed by the acquisition and removal of proteins and a marked drop in pH [22,23]. This process of phagosome “maturation” is comprised of a series of strictly coordinated membrane fission/fusion events between the phagosome and compartments of the endo/lysosomal network and culminates with the formation of the phagolysosome, a degradative organelle endowed with potent microbicidal properties [24,25,26] (Figure 2).

Engulfment of microbial prey is initiated at the plasma membrane of the macrophage where a vast repertoire of phagocytic receptors (e.g., Dectin-1, FcγR, and Mac-1) recognize the bacterial surface directly or indirectly through deposition of serum opsonins such as IgG or the complement protein C3b [27,28,29]. To facilitate the capture of phagocytic prey, macrophages, like dendritic cells, constitutively elaborate dynamic actin rich membrane protrusions that flail about probing the extracellular space [28,30,31]. Upon contact of the macrophage plasma membrane with the phagocytic target, receptors are engaged and cluster laterally along the plane of the plasma membrane to transduce signals to the interior of the cell triggering localized lipid remodeling and rearrangement of the actin cytoskeleton that underlies the membrane [32,33]. While the specific signaling molecules that are elicited downstream of distinct phagocytic receptors can vary, the requirement for actin remodeling in the engulfment process is absolute, as actin polymerization is required for the elaboration of pseudopodia that envelop the phagocytic target [34,35]. Incredibly, while actin polymerization proceeds at the leading edge of the pseudopod, F-actin is simultaneously depolymerized at the base of the forming “phagocytic cup”, presumably to allow for entry of newly formed cargo-containing phagosomes into the cytoplasm [21,36].

As previously alluded to and depicted in Figure 2 the nascent phagosome undergoes significant biochemical changes and follows a strictly choreographed maturation pathway [24]. Indeed, like the early endosome, the early phagosome can be defined by the acquisition of the small GTPase Rab5 that, in its active GTP-bound state, promotes phagosome remodeling [37,38]. Rab5 exerts its effects through the recruitment of numerous effector proteins including hVps34, the 3ʹ phosphatidylinositol kinase responsible for the local synthesis of phosphatidyl inositol 3-phosphate (PI(3)P) on the early phagosome [39,40]. Through a process coined “Rab conversion” the early phagosome acquires the small GTPase Rab7 with the concomitant loss of Rab5 as it matures to a late phagosomal stage [26,41,42]. Importantly, acquisition and activation of Rab7 is imperative for phagosome maturation as inhibition of this GTPase prevents further maturation and lysosomal fusion [43,44]. This is in part attributable to the Rab7-dependent recruitment of the protein RILP that links Rab7-positive endomembranes with the dynein-dynactin microtubule based motor complex that promotes phagosome displacement and lysosomal fusion [44,45,46]. While Rab7 and RILP are important, other unidentified phosphatidylinositol 3ʹ kinase (PI3K)-dependent processes that operate in parallel or downstream of Rab7 to mediate phagolysosome formation must also exist [26]. The late phagosome is also marked by the lysosome-associated membrane proteins 1 and 2 (LAMP-1 and LAMP-2), which are abundant transmembrane glycoproteins that were tacitly thought to function as scaffolds in the maintenance of lysosome integrity and acquired by phagosomes upon phagolysosome formation. In contradiction to this hypothesis it is now evident that LAMPs are, in fact, essential to the maturation process. Indeed, cells deficient for LAMP-1 and LAMP-2 produce phagosomes that fail to acquire Rab7 and do not acidify, implying that LAMP acquisition occurs prior to or concomitantly with Rab conversion [47]. In view of this, phagosomes marked with LAMPs are, in fact, maturing but by no means have they completed the process. This being stated, lysosomes are endowed with LAMPs so presumably some fraction of LAMP on the mature phagolysosome does indeed derive from lysosomes. Incredibly, during this entire process, despite that cellular components are obviously delivered to the maturing phagosome, its size remains constant. This phenomenon is attributed to the action of recycling pathways that allow for egress of membrane and retrieval of proteins (e.g., transferrin receptor, cation-independent mannose 6-phosphate receptor, and FcγR) from the maturing phagosome [48,49,50,51,52]. Ultimately the maturation process culminates with the formation of the phagolysosome, an inhospitable organelle where microbial growth is restricted and ingested bacteria are killed and degraded. This compartment can be defined by its marked acidity and its enrichment with a profusion of degradative enzymes including proteases, lipases, nucleases, phosphatases and glycosidases [53,54]. While a multitude of proteins comprise the lysosome their practical utility as *bona fide* lysosomal markers in the cell biological characterization of lysosomes and phagolysosomes is limited. As such fluorescent fluid phase tracers, such as indigestible dextrans, can easily be employed experimentally to mark lysosomes in living cells through pulse-chase approaches [55]. Through such dextran pulse chase approaches phagolysosomes can also be identified and therefore it can be determined experimentally whether a bacterium evades or resides within the phagolysosome where ingested microbes normally die [56,57,58,59]. Indeed, the potent microbicidal capacity of the phagolysosome is critical to immunity and is achieved through the concerted action of numerous antimicrobial effectors that are discussed below.

## 4. Phagosome Acidification

A hallmark of phagosome maturation is the progressive acidification of the phagosome lumen, where in the mature phagolysosome a pH of ~5.0 or less is achieved [23,60,61]. This drastic decrease in pH is catalyzed by the vacuolar ATPase, a H^+^ pump that extrudes H^+^ into the phagosome lumen from the cytosol at the expense of ATP [23,62]. In addition, the accumulation of H^+^ in the maturing phagosome is enhanced by the decreased passive permeability or “proton leak” of the limiting phagosomal membrane, which aids in the maintenance of an acidic pH [60,61]. Sustained acidification requires continuous H^+^ pumping by the v-ATPase however this is a highly electrogenic process that, in the absence of compensatory mechanisms, will be prohibited [54,63]. To dissipate inhibitory electrical potentials other ion transport processes, including cation efflux (e.g., Na^+^ and K^+^) and the inward flux of anions (e.g., Cl^−^), operate during phagosome maturation [54,64,65]. The importance of phagosome acidification must not be understated, as it is essential to the function of this organelle. Luminal acidification is not only a consequence of phagosome maturation but is also a prerequisite as indicated by the fact that the presence of weak bases (e.g., NH_4_Cl) or agents that specifically inhibit the function of the v-ATPase also arrest maturation [60,66,67]. Moreover, while phagosome acidification is in and of itself antimicrobial it is also intertwined with the function of other microbicidal and degradative processes in the phagosome and phagolysosome. For instance some lysosomal proteases, such as cathepsins, catalyze optimally at low pH while the metal transporter Slc11A1 (NRAMP-1, discussed below) that contributes to phagosomal nutrient limitation requires phagosomal H^+^ for its activity [68,69].

## 5. Reactive Oxygen and Nitrogen Species

It is well established that activation of the phagocyte NADPH oxidase (Nox2) is a vital innate immune mechanism. This is clearly evident in patients who suffer from Chronic Granulomatous Disease (CGD) who, because of genetic mutations in the genes encoding the NADPH oxidase, are profoundly susceptible to infection [70]. This is because the NADPH oxidase catalyzes the formation of the highly unstable super oxide (O_2_^−^) that sets in motion a variety of chemical reactions that generate noxious ROS that damage proteins, lipids and DNA [71]. While O_2_^−^ presumably contributes directly to bacterial killing, under the acidic conditions of the phagosome O_2_^−^ will rapidly dismutate into H_2_O_2_ or react with nitric oxide (NO) to form peroxynitrite (ONOO^−^), both of which are potent cell damaging agents [71]. Additionally, the liberation of transition metals such as iron (Fe) from proteins in the phagosome can result in Fenton chemistry and the production of hydroxyl radicals (OH^−^) that react non-discriminately with contents of the phagosome [71].

Activation of the NADPH oxidase is subject to strict spatio-temporal regulation and occurs in response to activating signals through phagocytic receptors (e.g., FcγR and/or Mac-1). The oxidase itself is comprised of the integral membrane proteins gp22^ph^°^x^ and gp90^ph^°^x^ that collectively form the flavocytochrome b_558_ responsible for the transfer of electrons from NADPH to molecular oxygen and the regulatory proteins p40^ph^°^x^, p47^ph^°^x^ and p67^ph^°^x^ that exist in a trimeric complex in the cytosol [72,73,74] and reviewed in [71]. In addition, the small GTPase Rac is required for optimal oxidase function [75,76]. Upon perception of activating stimuli the cytosolic subunits along with Rac1 associate with the flavocytochrome b558 to allow for the production of superoxide within the phagosome lumen. Incredibly, the production of O_2_^−^ can be detected in the phagocytic cup, even before fusion of the pseudopodia has occurred, indicating that assembly of this complex occurs very quickly [77,78]. Presumably then, this can be achieved because even at rest a significant fraction of the cellular flavocytochrome b558 resides at the plasma membrane in addition to intracellular vesicles that can be delivered to the phagosome [60,77,79].

While oxidase activation clearly contributes to the microbicidal functions of the phagosome, there exist important differences in oxidase activity between differentially polarized human macrophages [60]. Evidence of this comes from the classically activated M1 and alternatively activated M2 macrophage paradigm where M1 macrophages produce a robust oxidative burst that is sustained for ~1.5 h after phagosome sealing. This is in contrast to M2 macrophages, which are far less adept at producing ROS through NOX2 and do so only transiently [60,80]. These differences profoundly affect the biochemistry of the phagosome and likely account in part for their differential capacity to kill ingested microbes. Interestingly, ROS production in the phagosome is also intertwined with the luminal pH of the phagosome [81,82]. This is because robust production of O_2_^−^, and its subsequent dismutation to H_2_O_2_, consumes protons to such an extent that the phagosome alkalinizes [60,83]. In contrast, phagosomes lacking significant O_2_^−^ concentrations, because of only transient NADPH oxidase activity, rapidly acidify. Moreover, as acidification is required for phagosome maturation to occur, NOX2-dependent alkalization can, in fact, arrest phagosome maturation, a phenomenon that is indeed observed in human M1 macrophages [60]. Conceivably, the robust oxidative burst of classically activated M1 macrophages is intended to ensure that any microbial prey is deceased before attempting to process it in the phagolysosome.

In addition to NOX2-dependent production of oxygen radicals, macrophages, among other phagocytes, have the capacity to produce nitrogen-based radicals that can contribute to microbial killing. Moreover, these nitrogen-bearing compounds can elicit cell-signaling events to shape macrophage responses to infection. Nitric oxide (NO) radicals are produced when L-arginine and oxygen are converted to L-citrulline and NO, a reaction catalyzed by the protein nitric oxide synthase 2 (NOS2) [84]. Interestingly, NOS2 is not expressed in macrophages at “rest” however in response to appropriate inflammatory stimuli (e.g., IFN-γ and/or lipopolysaccharide) gene transcription is significantly enhanced [85]. It is important to emphasize that there exist significant differences between human and murine macrophages in terms of their capacity to produce NO however, NO production presumably contributes to the innate immune function of macrophages of both origins despite some contention (see [86,87,88,89] and the references therein).

When expressed, NOS2 appears to have a broad cellular distribution and has been localized to the cytosol, to undefined vesicular compartments, and to the cytosolic face of phagosomes [90,91,92,93]. Presumably from any of these locales NOS2-derived NO can diffuse and react with a profusion of intracellular components to elicit an effect. For instance, upon diffusion of NO into the lumen of the phagosome, NO may react with superoxide to form the highly reactive ONOO^−^ that can react chemically to modify proteins and DNA to intoxicate ingested microbes [94,95]. Additionally, NO can directly damage bacterial enzymes thereby compromising pathogen fitness and curtailing microbial growth during immune responses [95,96]. The antimicrobial effects of NO can also occur indirectly. Indeed, at non-toxic concentrations NO can modulate host cellular responses as evidenced by the NO-mediated activation of transcription factor Nrf2 that enhances the expression of the iron export protein ferroportin as part of the nutritional immune response of the macrophage (described below) [97].

## 6. Antimicrobial Proteins and Peptides

The lumen of the phagolysosome is an inhospitable environment and not surprisingly many successful intracellular pathogens (e.g., *M. tuberculosis*, *L. monocytogenes* and *L. pneumophila*) perturb host cell function to impede or alter conventional phagosome maturation (reviewed in [98]). Implicit in this is that the phagolysosome is a niche to avoid because it is a toxic compartment to microbes. The cathepsin family of cysteine proteases is perhaps the most extensively characterized group of lysosomal proteases and display a range of functions that shape the cellular immune response in macrophages. After phagocytosis some cathepsins have been identified as antimicrobial effectors that promote killing of phagocytosed pathogens. For instance, in the context of *S. aureus*, cathepsin L is a reported executor of non-oxidative killing of phagocytosed cocci ostensibly through direct proteolytic attack [99]. In contrast, cathepsin D may thwart immune evasion factors by degrading secreted bacterial effectors in the phagosome lumen as has been described for the pore-forming toxin listeriolysin O of *L. monocytogenes* [100]. Interestingly, cathepsins can also indirectly modulate host immunity by influencing the production of cytokines (e.g., IL-6 and IL-1β) from macrophages, which have more global effects on immune cell recruitment and function [99,101]. A vast repertoire of lysosomal hydrolases also exist that promote the degradative and killing capacity of the phagolysosome [53]. One such example is lysozyme, a well-known antibacterial protein that can be expressed and secreted by a variety of cell types, that catalyzes the hydrolysis of β1-4 glycosidic linkages between *N*-acetylmuramic acid and *N*-acetyl-d-glucosamine within peptidoglycan [102,103,104]. Importantly, compromised peptidoglycan integrity can promote instability and lysis of bacterial cells, which can presumably be amplified by the concerted action of antimicrobial proteins like human cathelicidin (hCAP-18/LL-37) in the phagolysosome [105,106]. Macrophage expression of cathelicidin can be induced via toll-like receptor signaling or through stimulation of the vitamin D receptor, which can profoundly influence the ability of macrophages to contain infection [107]. Indeed, LL-37 can play a direct role in microbial killing by promoting the disruption of bacterial membranes and its production can evoke autophagic responses, an important containment mechanism employed to combat intracellular infection by select bacterial pathogens and HIV [108,109]. Interestingly, LL-37 likely has a host of other immunomodulatory roles evidenced in part by its ability to elicit from macrophages (and other immune cells) the release of eicosanoids (e.g., leukotriene B4), antimicrobial peptides, and modulate leukocyte migration and function [110,111,112]. Phospholipases also comprise part of the macrophage's antimicrobial arsenal and exert their microbicidal effects by directly compromising bacterial membranes or promoting the production of immunomodulatory compounds. A prominent example of such an effector is the secreted group IIA secreted phospholipase A2 (IIA-sPLA2). This lipase, belonging to family of low-molecular-weight (~14 kDa) secreted PLA2) proteins, has potent antimicrobial activity against Gram-positive and Gram-negative bacteria [113,114]. Interestingly, in the absence of infection the serum concentration of IIA-PLA2 is maintained at low levels however as an acute phase protein its expression may increase by as much as ~1000 fold where it can participate in the antimicrobial response of the host [115,116]. Importantly, IIA-sPLA2 can promote the extracellular killing of bacteria or, as has been shown for neutrophils, contribute to the intracellular killing of *S. aureus* as it is taken up from the extracellular milieu during ingestion of bacteria [113,117]. Presumably, a similar phenomenon would occur during macrophage-mediated phagocytosis of *S. aureus*; however, since macrophages, as opposed to neutrophils, have the added capacity to express IIA-sPLA2, it is reasonable to speculate that IIA-sPLA2 is delivered to the mature phagosome in the macrophage.

## 7. The Role of the Macrophage in Nutritional Immunity

While the macrophage deploys an extensive repertoire of factors that are microbicidal, macrophages also enlist the activity of proteins that curtail microbial growth by limiting the availability of essential nutrients. The active sequestration of nutrients by the host, termed “nutritional immunity”, represents an important host defense strategy and successful pathogens like *S. aureus* have evolved nutrient acquisition systems that can circumvent the host response [118]. Perhaps the most extensively characterized mechanisms of nutritional immunity revolve around the sequestration of host Fe and Mn away from invading microbes. In the macrophage, the maturing phagosome becomes increasingly Fe- and Mn-deplete due to the extrusion of these elements from the phagosome lumen by the protein natural resistance-associated macrophage protein 1 (NRAMP-1) [69,119,120]. NRAMP-1 is an integral membrane protein that becomes enriched on maturing phagosomes as a result of lysosomal fusion and functions as an H^+^-dependent transporter of divalent cations [69,119]. Indeed, polymorphisms in the Slc11A1 gene (encodes NRAMP-1) that compromise functionality are associated with increased susceptibility to infection by intracellular pathogens (e.g., *M. tuberculosis* and *S. typhimurium*), emphasizing the importance of this protein in the nutritional immune response [121,122,123]. Upon extrusion of Fe from the phagosome into the cytosol Fe is rapidly sequestered by cytosolic chaperone proteins that deliver their Fe cargo to ferritin proteins for storage [124,125,126]. Presumably, while bound by ferritin, Fe is rendered inaccessible to microbes, however Fe can be liberated upon ferritin degradation in the lysosome [127,128]. Ostensibly, Fe released in this way is extruded from the phagosome by NRAMP-1 and is then exported from the cell by the integral membrane protein ferroportin (Fpn) [129,130]. Interestingly, the expression and function of Fpn is subject to multiple levels of regulation that are influenced by both infection and the demands of the host for iron [97,131,132,133]. When localized to the plasmalemma of the macrophage Fpn can extrude iron from the cytosol to the extracellular milieu, however during infection the ejection of cellular Fe stores may in fact promote growth of the invading pathogen. To circumvent this phenomenon hepcidin, an acute phase protein that is secreted from the liver, binds to surface expressed Fpn to induce its endocytosis, and subsequent degradation, thereby preventing Fe export [131,133,134]. Interestingly, upon perception of pathogen-associated molecular patterns through TLR signaling, macrophages can synthesize and secrete hepcidin, which will act in an autocrine and paracrine fashion to modulate Fe egress through Fpn [135]. While this hepcidin triggered response may be advantageous during an extracellular infection, in instances when macrophages harbor intracellular pathogens the sequestration of Fe to the interior of the cell may in fact enhance the likelihood that Fe will be obtained by the pathogen [136]. To compensate for this scenario down-regulation of Fpn at the macrophage plasmalemma can be overridden upon activation of iNOS as transcription of the Fpn gene is enhanced in response to NO [97].

While the aforementioned nutritional immunity proteins directly interact with divalent metals, the host protein lipocalin (Lcn) exerts its affect by interfering with bacterial siderophore mediated iron acquisition [137,138]. Indeed, Lcn binds selectively cathechol type siderophores (e.g., enterobactin or mycobactin) expressed by *Escherichia coli* and *M. tuberculosis*, respectively, sequestering Fe-laden siderophores from the bacteria. In macrophages Lcn can be induced through TLR-dependent or Fe-responsive signaling pathways and reportedly restricts intracellular growth of *S. typhimurium* [139,140].

While the concept of nutritional immunity relates primarily to mechanisms whereby the host restricts nutrient availability to curtail microbial growth, it is interesting that macrophages may actually harness the toxicity associated with high concentrations of certain transition metals (e.g., Zn and Cu) to effect metal-induced killing inside phagosomes [141]. Indirect evidence of this antimicrobial strategy emerges from transcriptomic analysis of phagocytosed *M. tuberculosis* where several P-type ATPases that function in the export of metals are induced [142]. Moreover, macrophages in response to infection or inflammatory cytokine stimulation can be made to induce expression Zn and Cu transport proteins such as Znt1 and ATP7A that operate at the limiting phagosomal membrane where they extrude these metals into the phagosome lumen [142,143]. More direct evidence that Cu and Zn ions can be employed as phagosomal "death metals" derive from studies where perturbation of metal ion transport into the phagosome or of the ability of ingested bacteria to detoxify these metals (*i.e.*, through mutagenesis of genes encoding metal export proteins) alters bacterial killing [143,144,145].

## 8. Macrophage Extracellular Traps (mETs)

NETosis, or the formation of extracellular traps comprised of host cell chromatin, was originally described as an innate immune mechanism employed by neutrophils to mediate capture and killing of microbes (reviewed in [146]). During netosis neutrophils eject their chromatin to the extracellular milieu where it is decorated with a host of neutrophil-derived antimicrobial proteins including, defensins, myeloperoxidase, lactoferrin and calprotectin [147,148,149]. More recently, it has been realized that multiple cell types, including macrophages, produce, in response to a variety of stimuli, related DNA-based structures that promote the entrapment and subsequent killing of bacterial pathogens including *S. aureus* [150,151,152,153]. Interestingly, the first description of macrophage extracellular traps (mETs), indicated that their formation was enhanced by perturbation of sterol synthesis in phagocytes [150]. Moreover, because inhibition of cholesterol synthesis impedes the ability of phagocytes to ingest and activate NOX2, extracellular trap formation may represent a compensatory response to maintain innate immunity. Despite these exciting findings the precise role of mETs in the innate immune function of the macrophage remains unclear as some literature suggests that despite mETs are decorated with macrophage-derived antimicrobial proteins (e.g., lysozyme), they are not microbicidal but rather function to ensnare extracellular bacteria so that other phagocytes can eradicate them [154].

## 9. *S. aureus* Evasion of Macrophage Defenses

*S. aureus* is endowed with an impressive armamentarium of virulence determinants that give this bacterium its pathogenic potential. The expression of such factors is governed by complex gene regulation pathways that are responsive to a plethora of environmental cues including, but not limited to, bacterial cell density, amino acid limitation, transition metal depletion, decreased pH, and oxidant production. Through integration of these environmental cues *S. aureus* can simultaneously coordinate expression of genes whose products will intoxicate immune cells, impede opsonization and phagocytosis, hinder complement fixation, detoxify ROS and RNS, repel antimicrobial protein attack, and extract host derived nutrients for growth. For a recent comprehensive summary of the literature related to the manipulation of host immune responses by *S. aureus* readers are directed to the recent review by Thammavongsa *et al.* [155]. Here we discuss the immune evasion strategies of *S. aureus*, specifically those related to evasion of macrophage-mediated responses.

## 10. Extracellular Intoxication of Phagocytes

The secretion of noxious proteins that intoxicate host cells represents but one of many strategies that *S. aureus* employs in order to counter host immune attack [156]. Indeed, professional phagocytes, such as macrophages, that are dead or dying are not phagocytic and will not be microbicidal [157]. *S. aureus* can elaborate a multitude of pore forming and membrane damaging toxins that evoke host cell death by promoting loss of membrane integrity and lysis of affected cells. These toxins, collectively termed leukocidins because they kill leukocytes include the bi-component leukocidins (e.g., LukAB (also known as LukGH), the pore forming toxin α-hemolysin (Hla), and a family of α-helical peptides called the phenol soluble modulins (PSMs) (e.g., α-PSMs) that demonstrate broad lytic activity towards many cell types (summarized in Table 1). While many of the toxins that belong to these groups are important in the pathogenesis of *S. aureus*, a detailed discussion of each is beyond the scope of this review, however there are several recent and thorough reviews that are dedicated to either the bi-component leukotoxins of *S. aureus* [158], the Hla protein [159], and the family of PSM peptides [160,161]. Nevertheless because the extracellular intoxication of host cells represents an important immune evasion strategy of *S. aureus*, a brief summary of how a few of these toxins affect macrophages is provided below.

For decades it has been appreciated that proteinaceous factors secreted by *S. aureus* can be cytotoxic towards mammalian cells. It is now realized that this cytotoxicity is due, in part, to the concerted action of as many as five distinct bi-component leukocidins (e.g., LukAB, LukED, HlgAB, HlgCB, and LukSF-PV) (see Table 1). Although there exist some important biochemical differences between individual leukocidins they appear to be unified in that they promote the disruption the cytoplasmic membranes of host leukocytes through the formation membrane spanning octomeric β-barrel pores [158,162,163]. This complex group of toxins is comprised of five distinct S subunits (e.g., LukA, LukE, HlgA, HlgC and LukS-PV) and four F subunits (e.g., LukB, LukD, HlgB, LukF-PV) that are secreted by *S. aureus* as soluble monomers and assemble into heterodimers at the surface of target cells [158,164,165]. Association with the host cell plasmalemma is driven by recognition of select surface receptors by the S subunit, which in turn recruits the corresponding F subunit to form receptor-associated heterodimers. Upon coalescence of four heterodimers into an octamer, a functional pore is formed, which will allow for the uncontrolled flux of ions across the cytoplasmic membrane. Interestingly, the LukAB toxin appears to deviate from this paradigm in that the LukAB heterodimer appears to assemble whilst in solution prior to interaction with the LukA receptor, CD11b, which is expressed at the surface of macrophages [166]. An important aspect of the leukocidin biology that must be emphasized is that membrane attack is mediated through engagement of specific surface receptors and, therefore, cells that are devoid of these receptors are immune to intoxication [164,167]. Conversely, the pattern of receptor expression between different cell types that can be engaged by any specific S subunit, will dictate which host cells are most susceptible to leukocidin attack (summarized in Table 1). For instance LukE binds directly to several chemokine receptors (e.g., CCR5, CXCR1, CXCR2, and DARC) however it is the expression of CCR5 on macrophages that renders these cells susceptible to LukED-mediated lysis [164,168,169]. In contrast, LukA is much less promiscuous and interacts exclusively with the CD11b subunit of the complement receptor Mac-1 that is expressed on both macrophages and neutrophils [167]. From these examples it is evident that *S. aureus* has the capacity to simultaneously launch a multipronged assault on macrophages through the production of different toxins (*i.e.*, LukAB and LukED) that target different receptors on macrophages (*i.e.*, CD11b and CCR5). Moreover it is reasonable to speculate that the dedication of such resources emphasizes the importance of phagocyte function in combating *S. aureus* infection and the selective advantage that can be gained by the bacteria upon phagocyte removal. Finally, the realization that the bi-component leukocidins of *S. aureus* usurp specific host receptors to elicit their biological effects has shed significant insight into the species specificity that these toxins can demonstrate as not all toxins recognize there cognate receptors if they are not of human origin (summarized in Table 1).

**Table 1:** *S. aureus* immune evasion factors that counter macrophage functions.

Evasion Strategy	Factor	Description	Ref.
Host cell intoxication	Leukotoxins		
	LukAB	Pore forming toxin; S subunit LukA engages CD11b subunit of Mac-1; targets macrophages and neutrophils of human origin	[167]
	LukED	Pore forming toxin; S subunit LukE engages CCR5, CXCR1/2, and DARC; targets macrophages, neutrophils, T-lymphocytes and red blood cells from many animal species	[164,168,169]
	LukSF-PV	Pore forming toxin; S subunit LukS engages complement receptors C5aR and C5aR2 of human and rabbit origin, targets neutrophils, monocytes and macrophages	[170,171]
	HlgAB	Pore forming toxin; S subunit HlgA engages CXCR1, CXCR2 and CCR2; targets neutrophils, monocytes and macrophages of human and murine origin with the exception that murine neutrophils are resistant to lysis	[172]
Host cell intoxication	Leukotoxins		
	HlgCB	Pore forming toxin, S subunit HlgC engages C5aR1 and C5aR2 to target neutrophils, monocytes and macrophages; demonstrates broad species specificity excluding mouse	[171,172]
	α-hemolysisn	Pore forming toxin; Utilizes host protein ADAM10 as receptor; Targets many cell types including macrophages of many origins including mice and humans	[173,174]
	α-PSMs	Small amphipathic peptides; broad lytic activity *in vitro*; may function as intracellular lysins	[175,176]
Avoidance of Phagocytosis	Opsonin Interference		
	Protein A and Sbi	Bind Fc region of IgG, occlude Fc region to prevent FcγR and C1q recognition	[177,178]
	Staphylokinase	Bacterial plasminogen activator; activates serine protease plasmin to promote degradation of complement and Ig	[179]
	Aureolysin	Secreted metalloprotease; degrades complement to prevent C3b opsonization	[180]
	Staphopain A/B	Secreted cysteine proteases; degrade complement thereby preventing opsonization	[181]
	V8	Secreted serine protease; degrades complement components and IgG	[181]
	Efb	Secreted bi-functional fibrinogen and C3b binding protein; Masks C3b on bacterial surface by promoting formation of a fibrinogen “shield”	[182]
	Capsule polysaccharide	Secreted polysaccharide polymer that encases the bacteria; shields bacterial surface from opsonins	[183,184]
	Complement inhibition		
	Cna	A collagen binding surface expressed protein; binds complement protein C1q; blocks C1q-dependent complement activation	[185]
	SCIN	Small secreted molecule; binds directly C3 convertase required for processing of C3 to C3a and C3b to inhibit convertase function	[186]
	Sbi	A cell wall associated and secreted protein; can recruit human plasminogen that is converted to plasmin to degrade C3; Can bind C3 products with the complement regulatory factor H to promote Factor I cleavage of C3b to inactive iC3b	[187,188]
	SdrE	Cell surface associated protein; binds Factor H recruiting it to the bacterial surface where it can act as a co-factor with Factor I to promote cleavage of C3b to iC3b	[189]
	ClfA	Cell-wall associated fibrinogen binding protein; binds Factor I that mediates cleavage of C3b to its inactive form iC3b	[190,191]
Evasion of macrophage anti-microbial defenses	Bacterial cell surface modification		
	OatA	Acetylates peptidoglycan to confer resistance to lysozyme	[192]
	DltABCD	Catalyze the incorporation of D-alanine into wall teichoic acids to reduce the negative charge of the bacterial cell surface; decreases binding of cationic antimicrobial peptides	[193]
	MprF	Catalyzes modification of negatively charged cytoplasmic membrane lipids by incorporating lysine residues to make the membrane less anionic; decreases binding of cationic antimicrobial peptides	[194]
	Eap, EapH1, EapH2	Secreted proteins that selectively inhibit the serine proteases neutrophil elastase, proteinase 3, and cathepsin G that are expressed by neutrophils; may function to inhibit serine proteases expressed by macrophages	[195]
	Resistance to ROS and RNS		
	SodA and SodM	Superoxide dismutases that detoxify ROS by catalyzing the conversion of superoxide into hydrogen peroxide	[196]
	KatA	A catalase that detoxifies hydrogen peroxide by catalyzing its breakdown into water and oxygen	[197]
	Msr	Methionine sulfoxide reductase; catalyzes the repair of methionine residues damaged by oxidation.	[198]
	Staphyloxanthin	A carotenoid expressed by *S. aureus* giving the cocci its golden pigmentation but can also, because of its molecular structure, act as an antioxidant; May also promote resistance to antimicrobial peptides as staphyloxanthin production decreases membrane fluidity	[199,200]
	Ldh1	A *S. aureus* specific lactate dehydrogenase that catalyzes the reduction of pyruvate to l-lactate with the concomitant oxidation of NADH to NAD^+^; helps maintain cellular redox balance in cells that are unable to respire because of nitric oxide mediated damage to electron transport chain proteins	[201]
	Hmp	Flavohemoprotein that scavenges NO to minimize damage to other *S. aureus* cellular components; up-regulated in response to nitrosative stress	[202,203]
Overcoming nutritional immunity	Divalent metal acquisition systems		
	Staphyloferrin A	Citrate based siderophore of *S. aureus*; extracts iron from host proteins to support staphylococcal growth; does not bind lipocalin	[204]
	Staphyloferrin B	Citrate based siderophore of *S. aureus*; extracts iron from host proteins to support staphylococcal growth; does not bind lipocalin	[205]
	Sst	Staphylococcal siderophore transport locus; encodes ABC transporter required for utilization of catechol siderophores and host derived, iron-binding stress hormones (e.g., norepinephrine)	[206]
	Fhu	The *fhuD1/2* and *fhuCBG* genes encode the receptors and permease, respectively, needed for utilization of hydroxymate type siderophores	[207,208]
	Isd	Isd proteins collectively allow *S. aureus* to bind hemoglobin, remove heme and shuttle it to the cytoplasm for use as an iron source	[209]
	MntABC and MntH	Transporters of Mn^2+^, required for growth under Mn^2+^ limited conditions (e.g., inside abscesses)	[210]

Abbreviations: DARC, Duffy antigen receptor of chemokines; SCIN, staphylococcal inhibitor of complement; Sbi, Staphylococcal binding of IgG; IgG; gamma immunoglobulin; SOD; superoxide dismutase; ROS; reactive oxygen species; RNS, reactive nitrogen species.

The Hla of *S. aureus* has been studied for decades and its importance in *S. aureus* pathogenesis is well established [159]. Indeed, Hla is another pore forming toxin in the arsenal of *S. aureus* that also targets macrophages and compromises cytoplasmic membrane integrity as evidenced by a recent report demonstrating that *S. aureus* growing in biofilms elaborate Hla, which acts synergistically with LukAB, to intoxicate macrophages, rendering them unable to phagocytose [157]. Hla assembles in the context of lipid bilayers into a β-barrel pore that is comprised of seven identical monomers [211]. Interestingly, Hla also interacts with a widely expressed host cell surface protein, the metalloproteinase ADAM10, which acts as a high-affinity receptor whose expression renders macrophages susceptible to intoxication [173,174]. Indeed, macrophages made deficient in ADAM10 are resistant to Hla, especially at low toxin concentrations [174]. In addition to Hla, *S. aureus* has the capacity to produce PSM peptides that can have lytic activity toward mammalian cells, however their effects have been most extensively characterized in the context of neutrophils. In contrast to the bi-component and Hla toxins, PSMs do not require cellular receptors to mediate their effects as they have an inherent propensity to insert into lipid bilayers owing to their amphipathic nature [212]. Despite their ability to interact with membranes without discrimination their effects on macrophages as extracellular toxins remain ill defined. In the seminal study by Wang *et al.*, [175] describing α-PSMs, it is revealed that while these secreted compounds elicited robust activation and destruction of neutrophils they elicited little response from macrophages. Moreover, their role as extracellular lytic toxins is unclear as lipoproteins found in blood and serum effectively neutralize secreted PSMs [213]. This may suggest that their cytolytic activity contributes to the escape of the phagocytosed bacteria, as has been demonstrated for neutrophils [176,213,214] and more recently suggested for macrophages [215]. In contrast, a recent report has demonstrated that *S. aureus* deficient in α-PSM production have a diminished capacity to evoke macrophage death through programmed necrosis [216] where presumably the α-PSMs are secreted by extracellular bacteria. Clearly, further study into the effects that PSMs have on macrophages is warranted.

At the cellular level, the outcome of pore formation due to Hla or leukocidins is similar in that they evoke inflammasome activation and the production of IL-1β [174,216,217,218]. For instance, it has recently been demonstrated that engagement of CD11b by LukAB activates NLRP3 containing inflammasomes and caspase-1, leading to production of the pro-inflammatory cytokine IL-1β, but only when acting at the cell surface [217]. Interestingly, when phagocytosed by macrophages, *S. aureus* reportedly kills infected cells from within the phagosome in a LukAB- and CD11b-dependent manner that is independent of caspase-1 activation [217]. Conceivably this disparity is explained by the fact that LukAB pores formed in the plasmalemma will allow for efflux of cytosolic K^+^ ions, a trigger of inflammasome activation, whereas LukAB pores formed in the limiting phagosomal membrane allows only for ion exchange between the phagosome lumen and cytosol without a net efflux of K^+^ from the cell [219]. Furthermore, recent evidence also demonstrates that membrane damaging toxins including LukAB and Hla elicit RIP1/RIP3/MLKL-dependent signaling and necroptotic death of macrophages addition to inflammasome activation [216,217]. Thus, multiple cell death pathways can act in concert during toxin-mediated attack, rendering macrophages unable to combat infection [216]. Importantly, there exists direct evidence that these phenomena are operational *in vivo* through murine infection models demonstrating the physiological importance of this immune evasion tactic [157,216]. In addition to delivering *S. aureus* synthesized proteins that directly intoxicate host cells this pathogen also reportedly utilizes host-derived molecules to evoke macrophage death. Indeed, using host-derived chromatin that is ejected to the extracellular milieu as a result of NET (and conceivably mET) formation, *S. aureus* catalyzes the production of toxic deoxyadenosine molecules that trigger macrophage death characterized by caspase-3 activation [220]. The *S. aureus* effectors that mediate this response are a secreted nuclease that that can degrade DNA and RNA to generate a pool of 3ʹ nucleotides that can be metabolized by the adenosine synthetase AdsA, which catalyzes the formation of deoxyadenosine molecules [220,221]. Previous studies had implicated both the secreted nuclease and AdsA in *S. aureus* pathogenesis and provided evidence to indicate that AdsA contributed to the evasion of phagocyte driven bacterial clearance however, the mechanism suppressing phagocyte function was attributed to the production of adenosine not deoxyadenosine [221]. Adenosine has potent anti-inflammatory and immunosuppressive effects and interestingly *S. aureus* usurps this host response to counter immunity during infection. Indeed, in murine blood *in vitro* and during murine infection *in vivo* AdsA-dependent increases in free adenosine can be measured and AdsA-deficient staphylococci are attenuated relative to their wild-type counterparts [221]. Biochemical characterization of AdsA from *S. aureus* has revealed that, in addition to the synthesis of deoxyadenosine as described above, AdsA can also catalyze the production of adenosine from ATP, ADP or AMP precursors [220,222]. The immunosuppressive effect of adenosine requires perception by host cells, which is mediated by four distinct receptors A1, A2A, A2B and A3, all of which can be expressed by macrophages among other cell types [223,224]. Importantly, in the context of the macrophage, alveolar macrophages exposed to adenosine, or AdsA competent *S. aureus*, demonstrate decreased production of the type 2 secretory phospholipase A2 and have a reduced capacity to phagocytose staphylococci in the airways of experimental animals [225]. Thus macrophages are indeed sensitive to the function of AdsA, which is a versatile enzyme and virulence determinant of *S. aureus* that can perturb macrophage function through production of adenosine and/or the induction of apoptosis.

## 11. Evasion of Complement and Opsono-Phagocytosis

Serum is enriched with an abundance of immunity related proteins that exert their functions by promoting the direct killing of microbial invaders or by marking microbes to make them recognizable by phagocytic receptors. For instance the pentraxins (e.g., Pentraxin 3) and immunoglobulins (e.g., IgG) can latch onto microbial surfaces and can engage the well-characterized Fc gamma receptors to mediate engulfment of opsonized targets by macrophages. Moreover, immunoglobulin and pentraxin deposition onto microbial surfaces can also provide a platform on which the processing of complement proteins can be initiated. Complement activation is a tightly regulated process that may proceed through three distinct pathways termed the alternative pathway (AP), classical pathway (CP), and lectin-dependent pathway (LP), however each pathway converges on the complement protein C3 to generate its bioactive components C3a and C3b [226]. Importantly, C3b is a ligand for the phagocytic receptor Mac-1 and upon its deposition onto reactive surfaces (e.g., bacteria) it marks targets for phagocytosis. Interestingly, C3b is also required for the formation of C5a and membrane attack complex, which act as a potent mediator of inflammation and forms lytic pores in bacterial membranes, respectively. While further description of these important immunity related molecules are beyond the purview of this review, we direct interested readers to recent reviews that are dedicated to the functional description of immunoglobulins [227], the pentraxin proteins [228,229], and the complement system [226,230]. Nevertheless it is clear that opsonization of bacteria with serum proteins and the fixation of complement represent formidable barriers that must be overcome in order for *S. aureus* to establish infection. Not surprisingly, *S. aureus* can produce a profusion of factors that impede opsonization and impair activation of the complement system. Some salient examples of *S. aureus* effectors that serve this function are discussed below.

## 12. Evasion of Opsono-Phagocytosis

Phagocytic receptors can broadly be categorized by whether they are non-opsonic (e.g., Dectin-1 and CD36) and directly recognize chemical structures displayed on microbial surfaces or whether they are opsonic (e.g., FcγRIIA and Mac-1) and indirectly recognize phagocytic targets through surface bound IgG or complement C3b (reviewed in Flannagan *et al.*,)[19]. While evasion of non-opsonic phagocytic receptor recognition by *S. aureus* has not, as of yet, been described, several factors have been identified that interfere with IgG and C3b opsonization to render the bacteria less palatable to the opsonic receptors expressed by macrophages.

Protein A, encoded by the *spa* gene, is a well characterized cell wall anchored protein that binds to the Fc region of IgG rendering it inaccessible to FcγR thereby preventing IgG-dependent phagocytosis [231,232]. Moreover, protein A binding of Fc can hinder the classical pathway of complement activation as the Fc of IgG is rendered inaccessible to the protein C1q that will ultimately reduce deposition of C3b on the bacterial surface [233]. Importantly, mutagenesis of the *spa* gene leads to attenuation of *S. aureus* pathogenesis in animal models of infection and allows for *S. aureus* to be more efficiently ingested by professional phagocytes [231,234,235]. Presumably these outcomes are attributable to a combination of increased FcγR-mediated and Mac-1-mediated phagocytosis. Interestingly, protein A can also be released to the extracellular milieu where it can have far reaching affects as a super antigen that modulates B lymphocyte function and adaptive immunity in animals [236], however further discussion on this topic this is beyond the scope of this review. An additional IgG binding protein, the Staphylococcal binder of IgG (Sbi) protein, is also expressed by most *S. aureus* strains as a cell wall-associated and a secreted protein, that akin to protein A, binds to the Fc domain of IgG to exert effects that are similar protein A [178,237,238]. Interestingly, while protein A and Sbi recruit IgG to the bacterial surface, albeit in a non-functional orientation, an alternative strategy employed by *S. aureus* to limit opsonization is simply to degrade opsonins within the local vicinity of the bacteria. Several secreted effectors, including staphylococcal proteases can exert this effect and *S. aureus* can also usurp host proteases to fulfill this function [179,180,239]. Indeed, the protein staphylokinase, a bacterial encoded plasminogen activator that converts host plasminogen to the active serine protease plasmin, can activate plasmin at the bacterial cell surface where it can catalyze the degradation of IgG and C3b [179].

While the aforementioned molecules impede opsonization of *S. aureus*, the bacteria also deploy a recently described failsafe mechanism that effectively shields bacterial-associated opsonin from phagocytic receptors on macrophages and neutrophils. During infection it is reasonable to assume that there is a fraction of C3b that is deposited on *S. aureus*, however, through recruitment of host fibrinogen the bacteria are able to encase themselves in a proteinaceous shield that masks the surface bound opsonin [182]. This effect, mediated by the secreted *S. aureus* effector protein Efb (extracellular fibrinogen binding protein), occurs when the C-terminus of Efb engages *S. aureus*-bound C3b while simultaneously binding the serum protein fibrinogen [182]. Clearly from the examples described above and in Table 1 it is clear that evasion of opsono-phagocytosis represents an important strategy employed by *S. aureus* to enhance its pathogenic potential.

## 13. *S. aureus* Inhibition of Complement Activation

As alluded to above, the complement system represents an import facet of the innate immune response and, not surprisingly, *S. aureus* encodes in its genome a profusion of effector molecules that counter complement-dependent responses (summarized in Table 1). Despite its importance a detailed discussion of complement evasion is beyond the scope of this review as many of the effects are not specific to macrophages and readers are directed to several recent comprehensive reviews dedicated to this topic [240,241]. This being stated and as is indicated above, inhibition of complement activation will undoubtedly diminish the production of the opsonin C3b thereby impairing opsono-phagocytosis through phagocytic complement receptors (e.g., Mac-1) expressed on phagocytes like macrophages. Additionally, and although not specific to macrophages, complement activation also produces the pro-inflammatory anaphylotoxins C3a and C5a that can indeed modulate macrophage function, among other cells. Macrophages express specific receptors that recognize the anaphylotoxins that when detected alter the macrophages state of activation, their ability to secrete various cytokines, and their microbicidal properties [157,242,243]. Interestingly, *S. aureus* growing in biofilms have the ability to skew the activation state of macrophages where they adopt an anti-inflammatory M2 phenotype [244]. While this effect is undoubtedly attributable to many factors, it is reasonable to speculate that perturbation of complement activation and diminished C5a and C3a production contribute.

In the CP pathway of complement activation the protein C1q is required for the initiation of complement processing and C1q is a target of *S. aureus* to block CP activation. Indeed, Kang *et al.* demonstrated that the prototypical collagen-binding MSCRAMM protein Cna could bind directly to host protein C1q thereby disrupting generation of a functional C3 convertase [185]. In addition to Cna targeting C1q, *S. aureus* can also secrete a low molecular weight protein, SCIN (staphylococcal complement inhibitor), that specifically binds to and stabilizes C3 convertases that generate the bioactive components of C3, C3a and C3b [186,245]. In addition to these molecules several additional staphylococcal proteins including the IgG binding protein Sbi function to bind various complement proteins to block the formation or activity of the C3 and C5 convertases (summarized in Table 1) indicating evasion of complement is critical for the success of *S. aureus* in the host. Interestingly, manipulation of complement signaling pathways may represent a therapeutic approach that can be employed to overcome the local immunosuppressive effects that *S aureus* can have. As mentioned above *S. aureus* growing in a biofilm is able to skew macrophage activation to yield phagocytes that are less microbicidal [244]. Interestingly, it has been shown in experimental animal models that treatment of infections with agonists of the C5a receptor can encourage macrophages to overcome this immunosuppressive effect [244].

In summary *S. aureus* can deploy an impressive arsenal of anti-complement and anti-phagocytic effectors implying that the ability of *S. aureus* to evade the hostile environment of the phagolysosome is an important survival strategy that enables *S. aureus* to persist during infection.

## 14. Resistance to Phagolysosomal Killing and Host Antimicrobial Proteins

The notion that *S. aureus* is primarily an “extracellular pathogen” has receded, as there exists a significant body of literature demonstrating that *S. aureus* can withstand killing after being phagocytosed by professional phagocytes including neutrophils and macrophages [56,246,247]. While many intracellular pathogens such as *M. tuberculosis* or *L. monocytogenes* employ virulence factors that allow them to evade the hostile environment of the phagolysosome, several reports describe that in many cell types *S. aureus* resides within mature phagosomes that are enriched with the lysosome-associated membrane proteins (LAMPs), markers of mature phagosomes [56,215,248]. While LAMPs are indispensable for phagosome/lysosome fusion, their presence on the limiting phagosomal membrane is not a definite measure of phagolysosome formation. Recently, our laboratory has published experiments, employing fluorescent dextrans chased into lysosomes, that demonstrate the rapid translocation of MRSA strain USA300 to the mature phagolysosome in macrophages [56]. Importantly, the frequency of lysosomal dextran co-localization between phagocytosed *S. aureus* and phagocytosed inert IgG-opsonized beads is indistinguishable, indicating that MRSA, unlike other intracellular pathogens, does not perturb phagosome maturation [56]. In contrast to this and a significant body of literature [56,61,215,248], a recent report suggested that *S. aureus*-containing phagosome fails to acidify and does not undergo lysosomal fusion [249]. Ostensibly, these discrepancies can be attributed to differences in experimental design, variances in experimental methods, and disparity between *S. aureus* strains. Despite some discrepancy in the description of the *S. aureus*-containing phagosome, several studies are unified in their conclusion that macrophages fail to eradicate phagocytosed cocci, which eventually cause cell death [56,217,250]. Previously it has been suggested that *S. aureus* survives innocuously within undefined membrane-bound vacuoles in the macrophage cytoplasm for several days until such a time that intracellular bacterial replication is initiated and macrophage lysis ensues [250]. Interestingly, extended residence within these cytoplasmic vacuoles is reportedly dependent on the global regulator Agr and the pore-forming toxin α-hemolysin, although the mechanisms by which these factors would promote extended residence in a membrane-bound vacuole are, at present, undefined [250]. In contrast, recent reports have demonstrated that macrophages harboring live *S. aureus* succumb to the infection within hours of phagocytosis and that cell death is preceded by intracellular proliferation of *S. aureus* [56,215,217]. Moreover, it has been suggested that, in macrophages, phagosome escape and entry into the cytosol is a prerequisite for replication, implying a lytic factor produced by the bacteria must be active in this compartment [215]. Several toxins (e.g., α-hemolysisn, α phenol soluble modulins, δ-toxin, or LukAB) purportedly fulfill this role in a variety of cell types, however there is no consensus on what toxin is responsible for dissolution of the phagosomal membrane or whether phagosome escape in macrophages is truly an evasive mechanism employed by phagocytosed *S. aureus* [215,246,248,251,252]. For neutrophils it is generally accepted that production of LukAB and the α-PSMs promote phagocyte destruction from within [213,246,253]. On the contrary, evidence contradicting phagosome escape of *S. aureus* in macrophages comes from recent work employing single cell imaging analysis of infected macrophages. These analyses clearly revealed that ingested *S. aureus* commence replication while confined to phosphatidylserine-positive membrane-bound vacuoles that are enriched with the phagosomal marker LAMP-1 [56]. Obviously with sustained replication the limiting phagosomal membrane will surely burst, however exit from the phagosome and access to the cytosol does not appear to be needed for *S. aureus* replication in macrophages. Further investigation of the cell biology of the macrophage/*S. aureus* interaction will undoubtedly provide more mechanistic detail about how these bacteria are able to survive intracellularly, however several factors are known to contribute.

By virtue of the fact that *S. aureus* replicates intracellularly the bacteria must withstand the hostile environment of the phagolysosome. As mentioned above, lysozyme represents an important antimicrobial protein that can digest the glycosidic linkages in the peptidoglycan (PGN) of many bacteria. In contrast to lysozyme sensitive bacteria, the peptidoglycan of *S. aureus* is immune to lysozyme attack due to the fact that the bacteria express acetylated peptidoglycan, a modification catalyzed by the acetyl-transferase OatA [254]. While acetylation renders PGN resistant to lysozyme, in macrophages it may also help evade detection by the inflammasome thereby suppressing IL-1β production [255]. While residing in the neutrophil *S. aureus* endures membrane attack by host derived antimicrobial peptides (AP) such as cathelicidin [256] and, conceivably, similar events transpire in macrophages. To repel AP attack *S. aureus* can modify negatively charged cytoplasmic membrane lipids by incorporating the positively charged amino acid lysine and can modify cell wall teichoic acids by incorporating alanine residues. These modifications, catalyzed by the enzyme MprF and the gene products of the *dlt* operon, respectively, mediate resistance to APs by making the bacterial surface less anionic thereby diminishing electrostatic interactions with cationic APs [193,194]. Importantly, inactivation of the *dlt* and *mprF* genes renders *S. aureus* more susceptible to leukocyte killing and attenuates pathogenicity in models of infection [194,257]. Attack on *S. aureus* in the phagolysosome is also mediated by proteases such as cathepsin L [99]. Interestingly, in the context of neutrophils, *S. aureus* can secrete a group of related protease inhibitors that specifically target the serine proteases neutrophils elastase, cathepsin G and proteinase 3 to afford protection against protease attack [195]. As such it is tempting to speculate that these inhibitors might also function in the macrophage to perturb protease-dependent attack of ingested *S. aureus*.

As described above the profound acidity of the mature phagolysosome (~pH ≤ 5.0) is but another antimicrobial property of the mature vacuole that must be endured by bacteria to survive in this niche. *In vitro* studies profiling the transcriptome of *S. aureus* in response to organic and inorganic acid stress reveal that a profusion of genes are differentially regulated and one such gene that is up-regulated in response to acid stress encodes urease [258,259]. Urease catalyzes the hydrolysis of urea to form ammonia, that under conditions of acidity acts as a weak base consuming protons to raise the pH [259]. Proof of this principle is evidenced by the loading of recombinant urease into lysosomes that, when in the presence of urea, selectively and reversibly alkalinizes acidified lysosomes [260]. Presumably similar acid responsive genes would be induced by *S. aureus* in the phagolysosome where their products would contribute to acid tolerance of ingested bacteria.

## 15. *S. aureus* Resistance to Oxidative and Nitrosative Killing

Activation of the NADPH oxidase and the resulting oxidative burst occurs swiftly and kills bacteria with significant potency. Indeed, O_2_^−^ formation can be detected in the forming phagosome prior to vacuole formation and, as such, *S. aureus* must be at the ready to counter ROS in order to maintain viability [77,78]. The bacteria carry two distinct genes, *sodA* and *sodM*, that encode super oxide dismutases and a single catalase encoding gene, *katA*, that will function in the sequential conversion O_2_^−^ to H_2_O_2_ to H_2_O and O_2_, respectively [261,262]. Importantly, these staphylococcal genes have all been implicated in conferring resistance to the microbicidal effects of ROS [262,263,264]. To complement these ROS detoxifying enzymes *S. aureus*, as its name aureus would suggest, produces the carotenoid pigment staphyloxanthin, which gives the bacteria its golden pigmentation and confers resistance to ROS mediated killing [199]. Interestingly, the protective effects of staphyloxanthin may also extend to non-oxidative killing as carotenoid production reportedly decreases membrane fluidity thereby promoting resistance to antimicrobial peptide attack [200]. Complete resistance to ROS mediated damage is unlikely and, as such, systems involved in the repair of damaged staphylococcal proteins also contribute to ROS resistance. For instance, genes encoding methionine sulfoxide reductases (Msr) that repair oxidized methionine residues in proteins, are up-regulated in neutrophil phagosomes [198] and presumably also in macrophages competent for NOX2 activation. Interestingly, the transcriptional regulation of these ROS detoxifying enzymes is complex and influenced in part by the regulators MntR, PerR and Fur that are responsive to varying concentrations of Mn^2+^, H_2_O_2_, and Fe^2+^ respectively [265,266,267]. Presumably such environmental cues can modulate resistance of *S. aureus* to ROS in macrophages however much of what is realized about the resistance of *S. aureus* to phagocyte NOX2 killing is derived from the study of human neutrophils. While investigation of NOX2-dependent killing of *S. aureus* in human macrophages deserves attention important considerations must be taken into account. For instance, not all macrophages are created equally in terms of their capacity to mount an oxidative burst (*i.e.*, M-CSF-derived macrophages have a diminished capacity for NOX2) so the correct macrophages must be employed if meaningful answers are to be realized [60,80]. Moreover, even in macrophages where a potent burst occurs, it will be for a limited duration (<1.5 h) and therefore measurements of NADPH oxidase-derived ROS and observations made outside this window must be carefully interpreted [60,268].

Phagocytosed *S. aureus* must also detoxify the effects of iNOS-dependent NO^−^ production. Perception of NO^−^ is important for bacterial adaptation and *S. aureus* is particularly well equipped with genes that promote bacterial survival and growth in response to NO challenge *in vitro* [203,269]. Detection of NO is mediated by the two-component system SsrAB, which is required for maximal resistance to nitrosative growth restriction [203,269]. In addition, the Fe-responsive regulator ferric iron uptake repressor (Fur) is also required for maximal survival in response to NO^−^ [203]. Resistance to NO^−^ is mediated in part by the SsrAB regulated gene *hmp* encoding a flavohemoglobin that functions as an NO^−^ scavenger [202,203]. Interestingly, *S. aureus* also encodes a lactate dehydrogenase Ldh1 that is induced in response to NO^−^ independently of SsrAB, and is required for sustained growth in the presence of nitrosative stress [201]. NO^−^ has the undesirable affect on *S. aureus* where it perturbs metabolism by disrupting redox homeostasis. To circumvent this affect *S. aureus* employs Ldh1 to maintain the ability of the bacteria to regenerate NADH from NAD^+^ in the presence of NO^−^ [201]. Importantly, mutagenesis of *ldh1* produces *S. aureus* bacteria that have a diminished capacity to overcome NO-dependent growth suppression in macrophages and that are less virulent in murine models of bacteremia. These experiments highlight the presence of the NO^−^ driven innate immune response and the ability of the bacteria to counter this mechanism of attack [201].

## 16. Overcoming Nutritional Immunity

Microbial growth is suppressed in the host through the concerted action of effectors that sequester essential nutrients from invading bacteria. Despite this, *S. aureus* encodes in its genome several factors that can circumvent host-imposed nutrient restriction. Fe is but one example of an essential transition metal that is strictly sequestered to curtail microbial growth and, as mentioned above, host proteins such as NRAMP and ferroportin operate in the phagosome and at the plasma membrane, respectively, to shuttle iron away from invading microbes. In response to Fe depletion *S. aureus* can up-regulate the expression of several dedicated Fe acquisition genes whose products are required for full virulence in animal models of infection (for detailed reviews and the references there in see [118,270,271]). Moreover, upon exposure to human blood or serum *S. aureus* enhances transcription of nutrient acquisition genes indicating the mechanisms needed for Fe-acquisition by the bacteria should be readily available upon ingestion by macrophages [272]. While the role of each of these systems inside the macrophage has not been elucidated the phagosome should, in principle, be a severely Fe depleted niche in part due to NRAMP1 function. One Fe acquisition strategy employed by *S. aureus* is reliant upon production of siderophores, which are low molecular Fe-binding compounds of such high affinity that they strip Fe from host proteins [204,205,206]. *S. aureus* can synthesize and import two distinct citrate based siderophores staphyloferrin A (SA) and staphyloferrin B (SB) that may be particularly effective in the host because they will avoid detection by the host siderophore binding protein lipocalin. In addition to SA and SB *S. aureus* is capable of utilizing siderophores produced by other bacteria (xenosiderophores) and host-derived hormones such as norepinephrine that can also bind iron [206,273]. Import of xenosiderophores and norepinephrine is mediated by the staphylococcal siderophore transporter (Sst) and ferric hydroxamate uptake (Fhu) siderophore import systems that are also up-regulated when Fe is deplete [205,206,207,208]. In addition to siderophore based systems *S. aureus* may also deploy the iron regulated surface determinant genes (Isd) that comprise a high-affinity heme and hemoglobin transport system that enables *S. aureus* to acquire Fe from hemoglobin and heme [274]. As *S. aureus* is capable of intraphagosomal replication in murine macrophage cell lines and in primary human M-CSF derived macrophages [56], it seems reasonable to assume that one or more of these Fe-acquisition systems are indeed active and can support intracellular growth of phagocytosed *S. aureus*.

Manganese (Mn) is another trace element that is actively sequestered from invading microbes during infection [275]. This effect is mediated largely by the abundant neutrophil protein calprotectin however calprotectin expression can also be elicited in macrophages in response to cytokine stimulation and/or toll-like receptor signaling [276,277,278]. To overcome Mn sequestration and support microbial growth *S. aureus* employs Mn transporters encoded by the *mntABC* and *mntH* loci [210,267]. While the role of Mn transport by *S. aureus* replicating within macrophages has not been addressed it is reasonable to assume that Mn acquisition would be needed to support microbial growth and maintain SOD activity [264] in this niche.

As opposed to limiting the access of trace metals such as Fe and Mn to some bacteria, there exists some evidence to suggest that Cu^2+^ extrusion into the phagosome may represent another host antimicrobial response [143]. In this regard, it is interesting that, in addition to Fe acquisition genes, the P-type ATPase CopA, a copper efflux protein, is up-regulated by *S. aureus* in human blood and serum [272,279]. While it has yet to be determined whether macrophage phagosomes harboring *S. aureus* are indeed copper replete it is tempting to speculate that *S. aureus* may be primed to cope with phagosomal death metals.

## 17. Concluding Remarks

Clearance of bacteria from the human host occurs habitually, however, there exist select bacterial pathogens that represent formidable threats to human health and *S. aureus* is no exception. The success of *S. aureus* as a professional pathogen can be attributed to the fact that its genome encodes an armamentarium of immune evasion proteins and toxins that destroy host cells. Macrophages, like neutrophils, are important sentinels of immunity that make significant contributions to innate defenses but they also participate in adaptive immunity. Nevertheless, despite their capacity to control microbial infection they fail to eradicate *S. aureus*. While significant advances have been made towards understanding the molecular details of the interaction between *S. aureus* and the macrophage important questions remain. As such a more complete understanding of the antimicrobial defenses of the macrophage and the mechanisms that *S. aureus* employs to evade killing will be required to devise novel therapeutics that could render the bacteria more susceptible to phagocyte attack.
